# *Ythdf2* Ablation Protects Aged Retina From RGC Dendrite Shrinking and Visual Decline

**DOI:** 10.1111/acel.70107

**Published:** 2025-05-15

**Authors:** Fugui Niu, Gaoxin Long, Jian Zhang, Jun Yu, Sheng‐Jian Ji

**Affiliations:** ^1^ Department of Neuroscience School of Life Sciences, Southern University of Science and Technology Shenzhen Guangdong China

**Keywords:** aging, dendrite, m^6^A, retinal degeneration, visual acuity, YTHDF2

## Abstract

Aging‐related retinal degeneration and vision loss have been severely affecting the elderly worldwide. Previously, we showed that the m^6^A reader YTHDF2 is a negative regulator for dendrite development and protection of retinal ganglion cells (RGC) in mice. Here, we further show that conditional ablation of *Ythdf2* protects the retina from RGC dendrite shrinking and vision loss in aged mice. Additionally, we identify *Hspa12a* and *Islr2* as the potential YTHDF2 target mRNAs mediating these effects. Together, our results indicate that the m^6^A reader YTHDF2 regulates retinal degeneration caused by aging, which might provide important therapeutic potential for developing new treatment approaches against aging‐related vision loss.

## Graphical abstract

The m^6^A reader YTHDF2 regulates retinal degeneration caused by aging, which is mediated by its target mRNAs *Hspa12a* and *Islr2*.

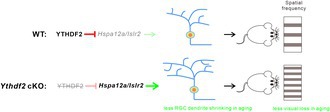

**Abbreviations**
cKO: conditional knockout
OMR: optomotor response
RGC: retinal ganglion cells

Vision loss and blindness in the elder are affecting hundreds of millions of people worldwide, which needs to be addressed as a public health issue with the aging global population (Blindness et al. 2021; Flaxman et al. 2017). Vision loss in old patients is mostly attributed to aging‐related macular degeneration, glaucoma, cataracts, and ocular complications of diabetes mellitus (Pelletier et al. 2016). The retina, as the fundamental structural tissue to encode and transmit visual signals into the brain, is organized by diverse cell types mediating the signal transduction cooperatively (Masland 2012). The degenerations in the aging retina are associated with such diseases as the progressive degeneration of photoreceptors in aging‐related macular degeneration and retinal ganglion cells (RGCs) degeneration in glaucoma (Fleckenstein et al. 2021; Weinreb et al. 2014). In addition, disease‐free vision decline is also relevant to structural and physiological changes in the retina, including RGC dendrite shrinking, retinal pigment epithelium degeneration, and photoreceptor dysfunction (Datta et al. 2017; Esquiva et al. 2017; Jackson et al. 2002; Owsley 2016; Samuel et al. 2011; Spear 1993). The dendrite arbor is the primary information receptive site in a neuron. The geometrical structure of its dendritic arbor determines the receiving region of input, synaptic density, numerous presynaptic partners, and certain physiological properties (Lefebvre et al. 2015). Rather than neuronal loss, alterations in the dendrite arbors, axonal collaterals, and synaptic density are anatomically detectable in the aged brains (Koch et al. 2021).

Previously we discovered that the m^6^A reader YTHDF2 negatively regulates dendrite development and injury of RGCs (Niu et al. 2022). The expansion of RGC dendrite arbors and more synapses in the inner plexiform layer after conditional knockout (cKO) of *Ythdf2* in the retina modestly improve the visual acuity of mice in an optomotor assay (Niu et al. 2022). In the glaucoma models, the m^6^A writers METTL3 and WTAP, and its reader YTHDF2, are upregulated, and the loss‐of‐function of YTHDF2 has a neuroprotective role (Niu et al. 2022; Qu et al. 2021). Besides, m^6^A modification and METTL3 expression are upregulated under hypoxic and diabetic stress, which governs retinal angiogenesis and pericyte dysfunction of retinal vascular complication (Suo et al. 2022; Yao et al. 2020). However, it remains unknown whether m^6^A modification and its reader YTHDF2 regulate the degeneration of RGCs in the aged retinas.

We kept *Six3‐cre* ^*+/−*^,*Ythdf2* ^*fl/fl*^ (*Ythdf2* cKO) and *Ythdf2* ^*fl/fl*^ control mice and had them grow to 23–25 months old, which is equivalent to approximately 70 years old for humans. We first checked the dendrite morphology of ipRGCs with anti‐melanopsin immunostaining in adult and aged mouse retinas. The dendritic area of melanopsin^+^ ipRGCs was significantly decreased in the aged control mice (23–25 months old, P24M) compared with adult control mice (3.5 months old, P3.5M) (a1 versus a3 in Figure 1a, and quantification in Figure 1b), which is consistent with the previous studies (Samuel et al. 2011).

**FIGURE 1:**
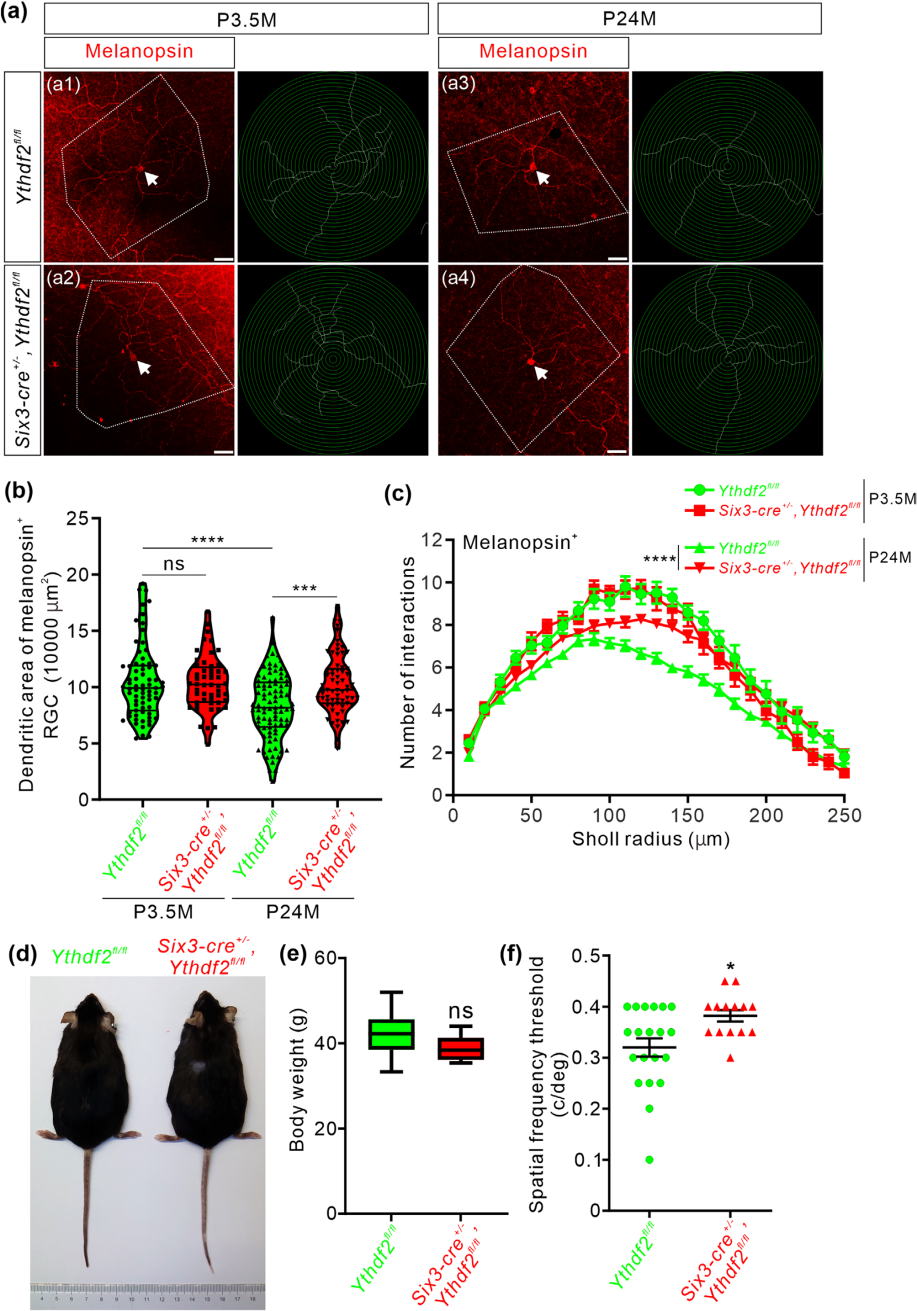
*Ythdf2* ablation protects aged retina from RGC dendrite shrinking and visual decline. (a) Representative images of wholemount immunostaining of adult (3.5 months old, P3.5M) and aged (23–25 months old, P24M) mouse retina using a melanopsin antibody. The RGCs of interest were pointed out with arrowheads. Areas of the convex polygon joining the outermost distal tips of each terminal dendrite were circled using white dotted lines. Dendrite traces were drawn for the corresponding RGCs shown. Scale bar: 50 μm. (b) Quantification of dendritic area of melanopsin^+^ ipRGCs in adult and aged mice shown in (a). Aged *Ythdf2* cKO mice (*Six3‐cre* ^*+/−*^,*Ythdf2* ^*fl/fl*^) maintain significantly larger dendritic areas compared with age‐matched control mice (*Ythdf2* ^*fl/fl*^). At least 3 mice were analyzed for each genotype and each stage. Data are represented as violin plots: *n* = 71 RGCs for control at P3.5M, *n* = 52 RGCs for cKO at P3.5M, *n* = 89 RGCs for control at P24M, *n* = 67 RGCs for cKO at P24M; control at P3.5M versus control at P24M, ****p = 1.33E‐05; control at P3.5M versus cKO at P3.5M, *p* = 0.99; control at P24M versus cKO at P24M, ***p = 1.30E‐04; ns, not significant; by Tukey's multiple comparisons test. (c) Quantification of dendrite branching of melanopsin^+^ ipRGCs in adult and aged mice shown in (a) using Sholl analysis. Numbers of interactions are similar between cKO and control mice at P3.5M and are significantly greater in cKO mice than control at P24M. At least 3 mice were analyzed for each genotype and each stage. Data are mean ± SEM: Control at P24M (*n* = 89 RGCs) versus cKO at P24M (*n* = 66 RGCs), ****p = 4.02E‐12, by Tukey's multiple comparisons test. (d) The aged *Ythdf2* cKO mice show similar body size as the control mice. (e) Quantification of body weight of the aged *Ythdf2* cKO and control mice. Data are represented as box and whisker plots: *p* = 0.18 (*n* = 10 control, *n* = 7 cKO; all male); ns, not significant; by unpaired Student's *t*‐test. (f) The aged *Ythdf2* cKO mice demonstrate better visual acuity. Quantification data are mean ± SEM: *p = 0.012 (*n* = 20 control, *n* = 14 cKO; all male); by unpaired Student's *t*‐test.

Although the dendrite branching of ipRGCs appeared denser in the young *Ythdf2* cKO mice compared to the young control mice at P20 (postnatal 20 days old) (Niu et al. 2022), there are no significant differences in dendritic area or branching in the adult *Ythdf2* cKO mice compared with control at P3.5M (a1 versus a2 in Figure 1a, and quantification in Figure 1b,c). These data suggest that the developmental phenotype of *Ythdf2* cKO in RGC dendrites is not maintained to adulthood.

Next, we continued to examine the dendrite morphology of ipRGCs in the aged *Ythdf2* cKO and control mice at P24M. The dendritic area of ipRGCs was significantly larger in the aged *Ythdf2* cKO mice compared to the age‐matched controls (Figure 1a,b). The ipRGCs of the aged *Ythdf2* cKO mice exhibited more dendrite branches and higher complexity compared to the age‐matched controls (Figure 1a,c). All these results suggest that the shrinking of dendrite area and complexity of ipRGCs caused by aging is dramatically alleviated in the aged *Ythdf2* cKO mice.

The aging‐related declines in spatial contrast sensitivity and visual acuity are attributed to neuronal changes, such as degeneration of RGC dendrites or axons (Samuel et al. 2011). The better RGC dendrite maintenance in the aged *Ythdf2* cKO mice inspired us to further explore whether the visual responses of the aged *Ythdf2* cKO mice were improved or not. The aged *Ythdf2* cKO mice showed similar body size and weight as controls (Figure 1d,e). We carried out the optomotor test on these aged mice. The aged control mice revealed significantly decreased visual acuity with the spatial frequency threshold as 0.32 ± 0.018 c/deg (Figure 1f), compared with the young control mice measuring 0.43 ± 0.0085 c/deg (Niu et al. 2022). However, the aged *Ythdf2* cKO mice showed significantly better visual acuity (0.38 ± 0.011 c/deg) compared with the aged control (Figure 1g). These data suggest that the ablation of *Ythdf2* in the retina improves the visual acuity of the aged mice.

By proteomic analysis and anti‐YTHDF2 RNA immunoprecipitation sequencing, we have identified *Hspa12a* and *Islr2* as the YTHDF2 targets in a glaucoma model (Niu et al. 2022). We further investigated whether *Hspa12a* and *Islr2* mediate RGC dendrite shrinking in the aged retinas. We first performed RT‐qPCR to check the expression levels of *Hspa12a* and *Islr2* by comparing the aged *Ythdf2* cKO and control retinas. Upregulation of their expression levels was detected in the aged *Ythdf2* cKO retina (Figure 2a), implying that the improved visual function in the *Ythdf2* cKO mice is likely mediated by the neuroprotective YTHDF2 targets *Hspa12a* and *Islr2*. Next, we continued to explore this pathway in the normal aging process. We found that the expression of *Mettl14* and *Ythdf2* was upregulated in the aged mouse retina compared with the young adults, although *Mettl3* expression was not changed (Figure 2b). In line with this, *Hspa12a* and *Islr2* mRNA levels were downregulated with aging (Figure 2c). The upregulation of the m^6^A writer *Mettl14* and reader *Ythdf2* in the aged retinas might account for the downregulation of *Hspa12a* and *Islr2*.

**FIGURE 2:**
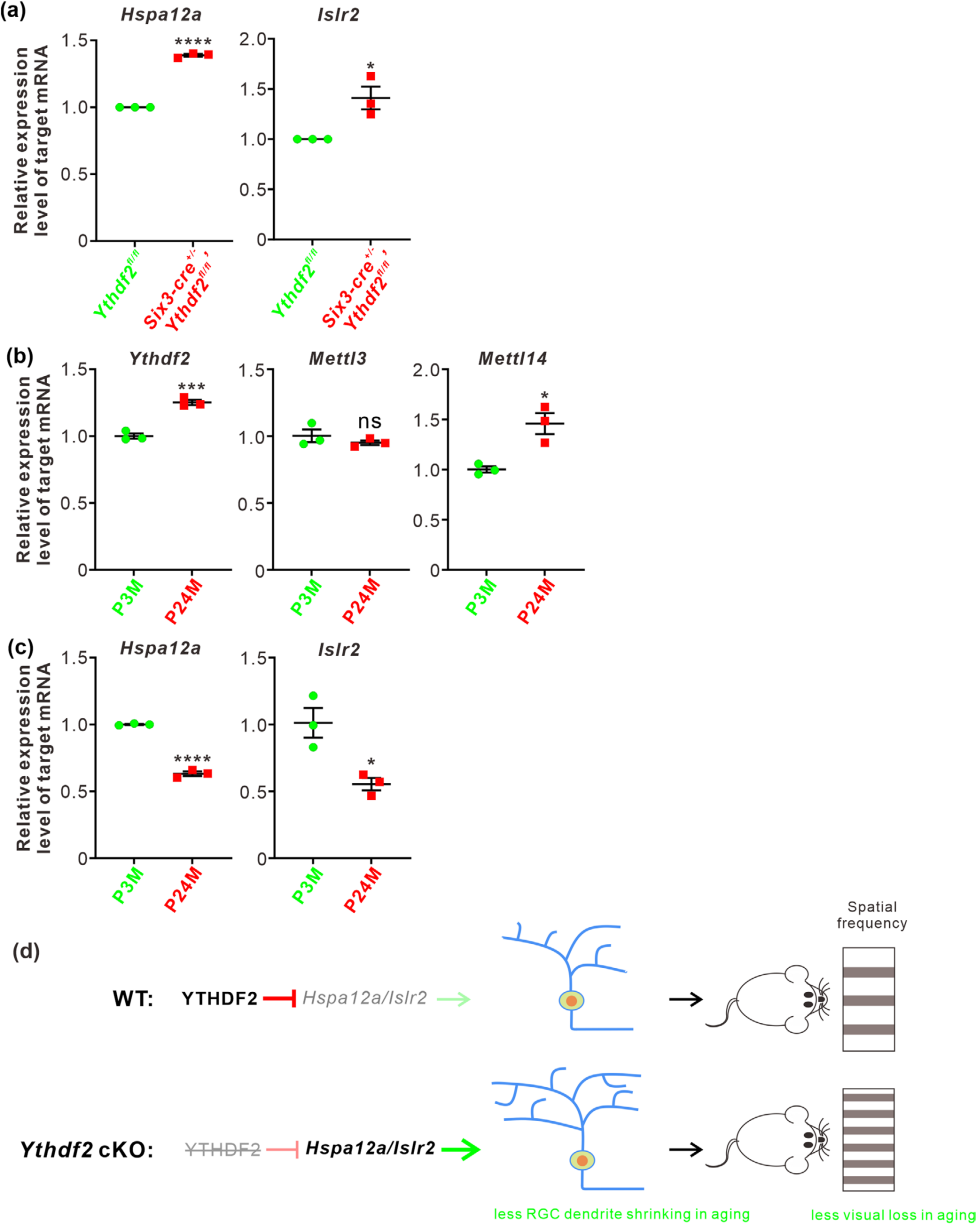
*Hspa12a* and *Islr2* are the YTHDF2 targets in the aged retinas. (a) Upregulation of YTHDF2 target mRNAs *Hspa12a* and *Islr2* in P24M *Ythdf2* cKO retina compared with control by RT‐qPCR. Data are mean ± SEM and are represented as dot plots (*n* = 3 replicates): ****p = 2.97E‐06 for *Hspa12a*, *p = 0.023 for *Islr2*, by unpaired Student's *t*‐test. (b) Upregulation of *Ythdf2* and *Mettl14* mRNA levels in the aged retinas. Data are mean ± SEM and are represented as dot plots (*n* = 3 replicates): ***p = 0.00081 for *Ythdf2*, *p* = 0.37 for *Mettl3*, *p = 0.014 for *Mettl14*; ns, not significant; by unpaired Student's *t*‐test. (c) Downregulation of *Hspa12a* and *Islr2* mRNA levels in the aged retinas. Data are mean ± SEM and are represented as dot plots (*n* = 3 replicates): ****p = 2.63E‐05 for *Hspa12a*, *p = 0.019 for *Islr2*, by unpaired Student's *t*‐test. (d) A proposed model for how YTHDF2 regulates aging‐related neurodegeneration in retina through its target mRNAs. Normally, YTHDF2 downregulates its target mRNAs *Hspa12a* and *Islr2* in the aged retinas, which leads to RGC dendrite shrinking and vision loss. Ablation of *Ythdf2* increases *Hspa12a* and *Islr2* levels in the aged retinas, which results in less RGC dendrite shrinking and less visual loss with aging.

Together, these data suggest that *Ythdf2* cKO protects the aged retina from aging‐related RGC dendrite shrinking and visual loss, possibly through avoiding downregulation of its neuroprotective targets, *Hspa12a* and *Islr2*.

In sum, in the previous work, we revealed that YTHDF2 is a negative regulator for dendrite development and injury of RGCs. Loss of function of YTHDF2 results in increased RGC dendrite branching, more synapses, improved visual acuity, and more resistance for glaucoma model (Niu et al. 2022). In this study, we further explored the function of the m^6^A reader YTHDF2 to mediate m^6^A modification in RGC degeneration and vision loss by aging. In the aged retinas, *Ythdf2* ablation disrupts the de‐stabilization of its target mRNAs and thus increases the levels of its target mRNAs including *Hspa12a* and *Islr2*, which results in less RGC dendrite shrinking and less visual loss (Figure 2d). The precise mechanisms of how the aging upregulates m^6^A modification and how the YTHDF2 target mRNAs protect neurodegeneration in retina still require further investigation. Nevertheless, the epitranscriptomic regulation through m^6^A modification and its reader proteins in gene expression can be evaluated as possible therapeutic targets for aging‐related vision decline.

## 1 Materials and Methods

### 1.1 Animals

*Ythdf2* ^*fl/fl*^ mice were reported previously (Yu et al. 2021), and *Six3‐cre* (Furuta et al. 2000) (The Jackson Laboratory, #019755) were from Jackson Laboratory. Genotyping primers are as follows: The first *Ythdf2*‐loxp site: 5′‐GCTTGTAGTTATGTTGTGTACCAC‐3′ and 5′‐GCAGCTCTGACTATTCTAAAACCTCC‐3′; the second *Ythdf2*‐loxp site: 5′‐CTCATAACATCCATAGCCACAGG‐3′ and 5′‐CCAAGAGATAGCTTTCCTAATG‐3′. *Six3‐cre* site: 5′‐CCTTCCTCCCTCTCTATGTG‐3′ and 5′‐GAACGAACCTGGTCGAAATC‐3′. All experiments using mice were carried out following the animal protocols approved by the Laboratory Animal Welfare and Ethics Committee of Southern University of Science and Technology.

### 1.2 Immunostaining

For anti‐melanopsin retinal wholemount staining, the process was described previously (Niu et al. 2022). All images were captured on a Zeiss LSM 800 confocal microscope with identical settings for each group in the same experiment.

### 1.3 RT‐qPCR

Total RNA was extracted from retinas with TRIzol Reagent (Life) and then used for reverse transcription by PrimeScript RT Master Mix (TaKaRa). Synthesized cDNA was performed with 2× ChamQ Universal SYBR qPCR Master Mix (Vazyme) on BioRad CFX96 Touch Real‐Time PCR system. Primers used for qPCR are as follows: mouse *Gapdh*: 5′‐TTGTCAGCAATGCATCCTGCACCACC‐3′ and 5′‐CTGAGTGGCAGTGATGGCATGGAC‐3′ (Niu et al. 2022); mouse *Ythdf2*: 5′‐GAGCAGAGACCAAAAGGTCAAG‐3′ and 5′‐CTGTGGGCTCAAGTAAGGTTC‐3′ (Niu et al. 2022); mouse *Hspa12a*: 5′‐GGGTTTGCACAGGCTAAGGA‐3′ and 5′‐TCTGATGGACGGTCAGGTCT‐3′ (Niu et al. 2022); mouse *Islr2*: 5′‐GAAGCTCCCTTAGACTGTCACC‐3′ and 5′‐CCCCATCGTGACTCCTGCTG‐3′ (Niu et al. 2022). Mouse *Mettl3*: 5′‐AACATCTGTGGCCCCTGAAC‐3′ and 5′‐AGGTGCATCTGGCGTAGAGA‐3′; mouse *Mettl14*: 5′‐TATGCTTGCGAAAGTGGGGT‐3′ and 5′‐CATCAGGCAATGCTCCTTTGT‐3′.

### 1.4 Optomotor Response (OMR) Assay

*Ythdf2* cKO and control mice aged about 24 months were applied for OMR assay as previously reported (Niu et al. 2022). Using the Matlab program, 0.075, 0.1, 0.2, 0.25, 0.3, 0.35, 0.4, 0.45, and 0.5 c/deg (30s per direction of rotation) were used in the recording process. Mouse behaviors were analyzed in real time during the experiment and re‐checked with the video recordings. The clockwise maximum spatial frequency that could drive head tracking to the rotation was defined as left eye spatial frequency threshold, and the counterclockwise one was defined as right eye spatial frequency threshold. Finally, the spatial frequency thresholds of the two eyes for each mouse were recorded and analyzed.

### 1.5 Sholl Analysis

Sholl analysis based on anti‐melanopsin retinal wholemount staining was described previously (Niu et al. 2022).

### 1.6 Dendritic Area Quantification

Quantification of dendritic area followed the previously reported protocols (Milosević et al. 2009). Briefly, the area of the convex polygon joining the outermost distal tips of each terminal dendrite was quantified with traced melanopsin signals.

### 1.7 Quantification and Statistical Analysis

All experiments were conducted at a minimum of three independent biological replicates in the lab. Statistical analysis was performed using GraphPad Prism 9.0. When comparing the means of two groups, an unpaired *t*‐test was performed on the basis of experimental design. The settings for all box and whisker plots are: 25th–75th percentiles (boxes), minimum and maximum (whiskers), and medians (horizontal lines). Data for all other graphs are mean ± SEM. A *p* value less than 0.05 was considered statistically significant: *p < 0.05, **p < 0.01, ***p < 0.001, ****p < 0.0001.

## Author Contributions

Conceptualization: F.N., S.‐J.J.; methodology: F.N., G.L.; investigation: F.N., G.L., J.Z., J.Y.; data analysis: F.N., G.L.; manuscript writing: F.N.; manuscript revision: F.N., G.L., S.‐J.J.; funding acquisition: S.‐J.J.; resources: S.‐J.J.; supervision: S.‐J.J.

## Conflicts of Interest

The authors declare no conflicts of interest.

## Supporting information


Movies S1–S5.

## Data Availability

The data that supports the findings of this study are available in the Supporting Information of this article.

## References

[acel70107-bib-0001] Blindness, G. B. D. , and Blindness and Vision Impairment Collaborators; Vision Loss Expert Group of the Global Burden of Disease Study . 2021. “Causes of Blindness and Vision Impairment in 2020 and Trends Over 30 Years, and Prevalence of Avoidable Blindness in Relation to VISION 2020: The Right to Sight: An Analysis for the Global Burden of Disease Study.” Lancet. Global Health 9: e144–e160. 10.1016/S2214-109X(20)30489-7. 33275949 PMC7820391

[acel70107-bib-0002] Datta, S. , M. Cano , K. Ebrahimi , L. Wang , and J. T. Handa . 2017. “The Impact of Oxidative Stress and Inflammation on RPE Degeneration in Non‐Neovascular AMD.” Progress in Retinal and Eye Research 60: 201–218. 10.1016/j.preteyeres.2017.03.002. 28336424 PMC5600827

[acel70107-bib-0003] Esquiva, G. , P. Lax , J. J. Pérez‐Santonja , J. M. García‐Fernández , and N. Cuenca . 2017. “Loss of Melanopsin‐Expressing Ganglion Cell Subtypes and Dendritic Degeneration in the Aging Human Retina.” Frontiers in Aging Neuroscience 9: 79. 10.3389/fnagi.2017.00079. 28420980 PMC5378720

[acel70107-bib-0004] Flaxman, S. R. , R. R. A. Bourne , S. Resnikoff , et al. 2017. “Global Causes of Blindness and Distance Vision Impairment 1990‐2020: A Systematic Review and Meta‐Analysis.” Lancet Global Health 5: e1221–e1234. 10.1016/s2214-109x(17)30393-5. 29032195

[acel70107-bib-0005] Fleckenstein, M. , T. D. L. Keenan , R. H. Guymer , et al. 2021. “Age‐Related Macular Degeneration.” Nature Reviews. Disease Primers 7: 31. 10.1038/s41572-021-00265-2. PMC12878645 33958600

[acel70107-bib-0006] Furuta, Y. , O. Lagutin , B. L. Hogan , and G. C. Oliver . 2000. “Retina‐And Ventral Forebrain‐Specific Cre Recombinase Activity in Transgenic Mice.” Genesis 26: 130–132. 10686607

[acel70107-bib-0007] Jackson, G. R. , C. Owsley , and C. A. Curcio . 2002. “Photoreceptor Degeneration and Dysfunction in Aging and Age‐Related Maculopathy.” Ageing Research Reviews 1: 381–396. 10.1016/s1568-1637(02)00007-7. 12067593

[acel70107-bib-0008] Koch, S. C. , A. Nelson , and V. Hartenstein . 2021. “Structural Aspects of the Aging Invertebrate Brain.” Cell and Tissue Research 383: 931–947. 10.1007/s00441-020-03314-6. 33409654 PMC7965346

[acel70107-bib-0009] Lefebvre, J. L. , J. R. Sanes , and J. N. Kay . 2015. “Development of Dendritic Form and Function.” Annual Review of Cell and Developmental Biology 31: 741–777. 10.1146/annurev-cellbio-100913-013020. 26422333

[acel70107-bib-0010] Masland, R. H. 2012. “The Neuronal Organization of the Retina.” Neuron 76: 266–280. 10.1016/j.neuron.2012.10.002. 23083731 PMC3714606

[acel70107-bib-0011] Milosević, N. T. , D. Ristanović , H. F. Jelinek , and K. Rajković . 2009. “Quantitative Analysis of Dendritic Morphology of the α and δ Retinal Ganglion Cells in the Rat: A Cell Classification Study.” Journal of Theoretical Biology 259: 142–150. 10.1016/j.jtbi.2009.03.011. 19298830

[acel70107-bib-0012] Niu, F. , P. Han , J. Zhang , et al. 2022. “The m(6)A Reader YTHDF2 Is a Negative Regulator for Dendrite Development and Maintenance of Retinal Ganglion Cells.” eLife 11: e75827. 10.7554/eLife.75827. 35179492 PMC8906807

[acel70107-bib-0013] Owsley, C. 2016. “Vision and Aging.” Annual Review of Vision Science 2: 255–271. 10.1146/annurev-vision-111815-114550. 28532355

[acel70107-bib-0014] Pelletier, A. L. , L. Rojas‐Roldan , and J. Coffin . 2016. “Vision Loss in Older Adults.” American Family Physician 94: 219–226. 27479624

[acel70107-bib-0015] Qu, X. , K. Zhu , Z. Li , D. Zhang , and L. Hou . 2021. “The Alteration of M6A‐Tagged Transcript Profiles in the Retina of Rats After Traumatic Optic Neuropathy.” Frontiers in Genetics 12: 628841. 10.3389/fgene.2021.628841. 33664770 PMC7920991

[acel70107-bib-0016] Samuel, M. A. , Y. Zhang , M. Meister , and J. R. Sanes . 2011. “Age‐Related Alterations in Neurons of the Mouse Retina.” Journal of Neuroscience 31: 16033–16044. 10.1523/jneurosci.3580-11.2011. 22049445 PMC3238393

[acel70107-bib-0017] Spear, P. D. 1993. “Neural Bases of Visual Deficits During Aging.” Vision Research 33: 2589–2609. 10.1016/0042-6989(93)90218-l. 8296455

[acel70107-bib-0018] Suo, L. , C. Liu , Q. Y. Zhang , et al. 2022. “METTL3‐Mediated N (6)‐Methyladenosine Modification Governs Pericyte Dysfunction During Diabetes‐Induced Retinal Vascular Complication.” Theranostics 12: 277–289. 10.7150/thno.63441. 34987645 PMC8690932

[acel70107-bib-0019] Weinreb, R. N. , T. Aung , and F. A. Medeiros . 2014. “The Pathophysiology and Treatment of Glaucoma: A Review.” JAMA 311: 1901–1911. 10.1001/jama.2014.3192. 24825645 PMC4523637

[acel70107-bib-0020] Yao, M. D. , Q. Jiang , Y. Ma , et al. 2020. “Role of METTL3‐Dependent N(6)‐Methyladenosine mRNA Modification in the Promotion of Angiogenesis.” Molecular Therapy 28: 2191–2202. 10.1016/j.ymthe.2020.07.022. 32755566 PMC7545007

[acel70107-bib-0021] Yu, J. , Y. She , L. Yang , et al. 2021. “The m(6) A Readers YTHDF1 and YTHDF2 Synergistically Control Cerebellar Parallel Fiber Growth by Regulating Local Translation of the Key Wnt5a Signaling Components in Axons.” Advancement of Science 8: e2101329. 10.1002/advs.202101329. PMC8596126 34643063

