# Supplementary material for: Ythdf2 Ablation Protects Aged Retina From RGC Dendrite Shrinking and Visual Decline
Source: Aging Cell. 2025 May 15;24(7):e70107. doi: 10.1111/acel.70107 (PMC12266746; doi:10.1111/acel.70107)
Supplement: Supplementary file 1 — Movies S1–S5. [file ACEL-24-e70107-s001.zip › MovieS1S5 caption.docx]

Movie 1, Representative movie of optomotor response (OMR) assay for aged control mice;

Movie 2, Representative movie of OMR assay for aged *Ythdf2* cKO mice;

Movie 3, Representative movie clip of OMR assay for aged control mice;

Movies 4 and 5, Representative movie clips of OMR assay for aged *Ythdf2* cKO mice.
